# Assessing Bone Health Status and Eggshell Quality of Laying Hens at the End of a Production Cycle in Response to Inclusion of a Hybrid Rye to a Wheat–Corn Diet

**DOI:** 10.3390/vetsci9120683

**Published:** 2022-12-08

**Authors:** Siemowit Muszyński, Kornel Kasperek, Sylwester Świątkiewicz, Anna Arczewska-Włosek, Dariusz Wiącek, Janine Donaldson, Piotr Dobrowolski, Marcin B. Arciszewski, Jose Luis Valverde Piedra, Dominika Krakowiak, Katarzyna Kras, Jadwiga Śliwa, Tomasz Schwarz

**Affiliations:** 1 Department of Biophysics, Faculty of Environmental Biology, University of Life Sciences in Lublin, 20-950 Lublin, Poland; 2 Institute of Biological Basis of Animal Production, Faculty of Animal Sciences and Bioeconomy, University of Life Sciences in Lublin, 20-950 Lublin, Poland; 3 Department of Animal Nutrition and Feed Science, National Research Institute of Animal Production, 32-083 Balice, Poland; 4 Department of Physical Properties of Plant Materials, Bohdan Dobrzański Institute of Agrophysics of the Polish Academy of Sciences, 20-290 Lublin, Poland; 5 School of Physiology, Faculty of Health Sciences, University of the Witwatersrand, Parktown, Johannesburg 2193, South Africa; 6 Department of Functional Anatomy and Cytobiology, Faculty of Biology and Biotechnology, Maria Curie-Sklodowska University, 20-033 Lublin, Poland; 7 Department of Animal Anatomy and Histology, University of Life Sciences in Lublin, 20-950 Lublin, Poland; 8 Department of Pharmacology, Toxicology and Environmental Protection, University of Life Sciences, 20-950 Lublin, Poland; 9 Department of Animal Genetics, Breeding and Ethology, Faculty of Animal Sciences, University of Agriculture in Kraków, 30-059 Cracow, Poland

**Keywords:** laying hens, rye, exogenous enzymes, eggshell quality, bone mineralization, osteoporosis

## Abstract

**Simple Summary:**

The eggshell provides physical protection of the internal components of the egg. Calcium, the main mineral forming eggshell, comes both directly from the diet but also from bones. Continuous bone demineralization may lead to osteoporosis and bone fractures. The main energy source in hens’ diet is corn, but also grains, like wheat or rye, can be introduced. However, due to the presence of non-starch polysaccharides, which cannot be utilized by poultry, absorption of nutrients might be reduced. The enzyme xylanase, supplemented in diets improves nutrient uptake. In this study, the effect of a modern hybrid rye included in a wheat–corn diet with xylanase supplementation on eggshell and bone quality in laying hens was examined. The results showed that hybrid rye can replace corn in hens’ diet without negative effects on eggshell quality or bone mechanical strength, but xylanase supplementation to these diets is recommended.

**Abstract:**

The objective of this study was to evaluate whether there are negative effects of the partial replacement of white corn with rye along with xylanase supplementation on overall bone quality, eggshell mineralization, and mechanical strength in laying hens. From the 26th week of life, ISA Brown laying hens were fed either a wheat–corn diet or a diet containing 25% rye, with or without xylanase. The experimental period lasted for 25 weeks, until birds reached their 50th week of age, after which bone and eggshell quality indices were assessed. Eggshell thickness and eggshell Ca content of eggs from rye-fed hens were improved by xylanase supplementation. No differences in the mechanical properties of the eggshells were observed between treatments, except for the diet-dependent changes in egg deformation. Rye inclusion had no effect on the mechanical properties of bone. Xylanase supplementation, irrespective of the diet, had a positive effect on bone strength and increased tibia Ca content, as well as the content of several microelements. Hence, hybrid rye combined with wheat can replace 25% of corn in layer diets without compromising shell quality or bone mineral content. Xylanase supplementation in these diets is recommended since its inclusion improves both bone strength and quality.

## 1. Introduction

Modern, hybrid strains of laying hens produce on average around 250 eggs a year. To maintain such effective egg production, the metabolism of laying hens is highly dependent on the availability and homeostasis of the minerals used in the formation of the eggshell, such as Ca and P [1]. Bones, especially medullary bone, formed on the endosteal surface of the long bones of maturating hens, are primary mineral storage organs. Calcium, phosphorus and other minerals required for eggshell production are successively released from medullary bone during the entire laying period [2,3]. However, mineral resorption is not limited to medullary bone, and a decrease in structural (cortical) bone mineralization during the laying period has been observed [4]. This could result in the development of osteoporosis, which leads to a decrease in bone mechanical strength and hens may suffer from bone breakage and other bone abnormalities, especially during the post-peak laying period [5]. Therefore, since hens require a substantial amount of calcium for eggshell formation, sufficiently mineralized bone throughout the whole laying cycle is crucial, not only to ensure the successful formation of properly mineralized eggshell, but also for the overall welfare and health of the hen.

The main physical function of the eggshell is to protect the internal components of the egg. Eggshell strength is characterized by its resistance to cracking, which can occur in the presence of external loads. Eggshell strength depends on the physical properties of the eggshell, characterized on a macroscopic level by its shape, weight, thickness and mineralization [6]. Eggshell quality is primarily influenced by genetic factors, laying rate, bird age, general health status, housing conditions, and nutrition [7,8,9]. Similarly, nutritional factors play a crucial role in the modulation of bone mineral homeostasis, influencing the mineralization and mechanical strength of the bones [10,11]. Corn is the main energy source in the diets of hens, and cereal grains like wheat, barley, or rye, have been successfully used as partial replacements for corn. However, a high-level of incorporation of grains in the diet leads to an increase in the content of non-starch polysaccharides (NSPs), which, among others, results in reduced absorption of nutrients, since NSPs cannot be broken down by non-ruminants’ enzymes [12,13,14]. To alleviate the negative effects of the inclusion of cereal grains in poultry diets and to improve nutrient uptake, the diets of laying hens are commonly supplemented with exogenous enzymes like endo-beta-1,4-xylanses, capable of degrading NSPs [15,16].

From an economical point of view, modern, high-yielding varieties of hybrid rye, which can be grown in a wider range of environmental conditions than wheat, seem to be attractive as a partial replacement of corn in poultry feeds [12,17]. However, there is a dearth of knowledge regarding the effects of substituting corn with hybrid rye and xylanase supplementation on bone and eggshell mechanical properties and mineralization which thus warrants investigation. Previously, we have shown the positive effects of xylanase supplementation on collagen maturity in the tibia of hens fed a wheat–corn diet with inclusion of 25% rye [18]. This indicated that the dietary inclusion of rye influenced bone homeostasis but did not provide any information regarding the effect of the diet on bone biomechanical properties or mineralization.

The current study aimed to fill this knowledge gap regarding the effects of modern hybrid rye, a new promising feed ingredient, on bone and eggshell quality. The objective of the present study was to investigate whether there are negative effects of replacing 25% of white corn with rye along with xylanase supplementation, on overall bone and eggshell mineralization and mechanical strength in laying hens during the post-peak laying period when the bone mineral reserves would be significantly required.

## 2. Materials and Methods

### 2.1. Experimental Design

The conditions under which the hens were housed are described in detail in a previous publication [18]. Briefly, during the pre-experimental period, 96, 17-week-old, commercial ISA Brown laying hens were housed with two hens per cage, under climate-controlled conditions, in wire-mesh floor cages, and were fed a standard commercial diet. At the 26th week of life the laying hens were randomly allocated to one of dietary treatment groups, each compromising of 12 replicate cages with 2 hens per cage. A 2x2 factorial arrangement was used, with hybrid winter rye cv. Brasetto inclusion (0% or 25%) and xylanase) supplementation (0 or 200 mg/kg of feed; declared activity of 1000 FXU/g) as factors. Xylanase (Ronozyme WX; DSM Nutritional Products Sp. z o.o., Mszczonów, Poland) was added to experimental diets “on top” of the feed formulation. All experimental diets (Table 1) [18,19] were formulated to meet or exceed the nutrient recommendations for hens [20]. Hens were fed their respective experimental diets until the 50th week of life, after which the procedures of sample collection took place.

### 2.2. Sample Collections and Preparations

Eggshell quality at the end of the 25-week-long experimental trial was determined using two eggs, randomly collected per cage, over two consecutive days. Collected eggs were stored for no longer than three days in a cold room (5 °C). For bone analysis, on the last day of the experimental trial, one hen from each replicate cage was randomly selected and slaughtered (*n* = 12 per group). Left tibias were carefully dissected out and stored at −20 °C until further examination.

#### 2.2.1. Basic Eggshell Quality Indices

After measurement of egg weight, egg shape index and volume were determined [6,24,25]. The eggshell thickness was measured using an ESTG-1, ultrasonic eggshell thickness gauge (Orka Food Technology Ltd., Ramat Hasharon, Israel) at 45° from the large end of the egg [26]. Eggshell percentage and eggshell density measurements were performed after eggshell mechanical testing; measurement of eggshell density was performed for oven-dried eggshell samples with a helium gas pycnometer [27].

#### 2.2.2. Eggshell Mechanical Properties

The measurements were performed with an Instron universal testing machine (Mini 55, American Instrument Exchange, Haverhill, MA, USA). The compression test was performed on one of two axes of the egg: polar plane (the loading axis through the length dimension), and equator plane (the transverse axis containing the width dimension). For each type of testing, one egg from each replicate cage was used. In both trials, eggs were compressed at a constant loading rate of 50 mm/min, until the eggshells cracked. The following mechanical parameters were determined using Origin software (ver. 2021, OriginLab, Northampton, MA, USA): the cracking force, defined as the force that caused the eggshell to crack; stiffness, defined as the slope of the first part of the load-displacement curve representing the elastic deformation of the eggshell [28]; firmness, defined as the ratio of cracking force to the absolute rate of deformation [25]; and the total work to eggshell cracking, defined as the area under the whole recorded load-displacement curve. For calculation of the structural properties of the eggshell of eggs compressed in the polar plane, formulas developed by Bain [29] were used. The eggshell Young’s elastic was calculated on the basis of measurement of eggshell thickness, egg radius of curvature and shape index, and stiffness, [30]. For calculation of fracture toughness, measurements of cracking force, radius of curvature, and eggshell thickness were used [28]. For calculation of eggshell Young’s elastic modulus, when eggs were compressed in the equatorial plane, formulas developed by Nedomova et al. [30], based on egg curvature measurements, were applied.

#### 2.2.3. Bone Densitometry

Bone mineral content (BMC) and bone mineral density (BMD) measurements of the tibias were performed using a DXA densitometer (Lunar, GE, Madison, WI, USA). The measurements were performed on the whole bone in the coronal plane.

#### 2.2.4. Measurements of Bone Mechanical Properties

A three-point bending test was performed on a Zwick Z010 universal testing machine (Zwick/Roell GmbH & Co., Ulm, Germany). Prior to the analyses, tibia weight and length were determined. During the bending test, the load was applied with a constant loading rate of 10 mm/min at the middle point of the bone diaphysis, in the cranial–caudal plane, until fracture. Geometric properties of the bone mid-diaphysis (cortical index, cross-sectional area, cross-sectional moment of inertia) were calculated on the basis of measurements of diameters of the bone diaphysis cross-section [31]. Mechanical properties of the bone (yield load, ultimate load, stiffness, elastic energy and work to fracture) were read from force-displacement data using Origin 2021 software [31]. Bone material properties (yield and ultimate stress, Young’s modulus, yield and ultimate strain) were also calculated based on the data recorded during the three-point bending tests and the performed measurements of tibia mid-diaphysis cross-sectional geometry [31].

#### 2.2.5. Measurements of Bone and Eggshell Mineral Phase

After mechanical testing, the bone marrow was removed, bones were crushed and defatted in a chloroform–methanol (2:1, *v*:*v*) solvent system. To determine the ash percentage defatted bone samples were dried at a temperature of 105 °C for 24 h and finally ashed in a muffle oven at a temperature of 500 °C for 24 h. Eggshell samples (taken from one egg per replicate cage) were treated in a similar manner, except for the defatting procedure. The chemical composition of the bones and eggshells’ mineral phase was determined in the ashed samples using ICP-OES spectrometry (iCAP 6500, Thermo Scientific, Waltham, MA, USA).

### 2.3. Statistical Analysis

The data were analyzed using a general linear model (GML) in Statistica software (v. 13.3, TIBCO Software Inc., Palo Alto, CA, USA) with rye inclusion and xylanase supplementation as the main factors and with the interaction of the main factors added to the model. A cage was considered an experimental unit. Where necessary (external properties of eggshell), the values of each measured trait of an individual egg in a replicate cage were averaged per cage prior to statistical analysis. The data were checked for normal distribution using the Shapiro–Wilk test, and then the Levene’s test was applied to test homogeneity of variances. As all variables passed the normality and variance homogeneity tests (*p* > 0.05), they were subjected to ANOVA analysis with a Tukey’s HSD as a post-hoc test. For all tests, a *p* < 0.05 was established as statistically significant.

## 3. Results

As reported in a previous paper [21], 25% inclusion of rye grain did not negatively influence basal laying performance indices (egg weight, daily mass of egg, daily feed intake by hens or feed conversion ratio) or basal integral egg quality indices (Haugh unit, yolk share) during the whole (25–50th week of age) experimental laying cycle. Eggs collected at the end of the experimental period were also analyzed for more detailed yolk and albumen quality [19]. The 25% rye inclusion had no significant effect on most of the examined egg physical properties, however, it increased yolk water content and decreased yolk fat content and had a beneficial effect on the yolk fatty acid profile, increasing the sum of mono- and polyunsaturated fatty acids and decreasing the omega 6:3 ratio [19].

### 3.1. Eggshell Properties

There was no effect of treatment on egg weight, volume or shape index at the end of the study (*p* > 0.05, Table 2). However, dietary inclusion of rye resulted in a decrease in eggshell percentage (*p* < 0.01). The eggshell thickness observed in eggs from hens in the rye-fed group deprived of enzyme supplementation was significantly lower than that observed in the other groups (*p* < 0.05). The inclusion of xylanase decreased eggshell density in the wheat–corn group and increased eggshell density in the group fed the diet with rye inclusion (*p* < 0.001).

The composition of the eggshell mineral phase is presented in Table 3. Eggshell Ca content was increased in the rye–corn–wheat group and decreased in the corn–wheat group following xylanase supplementation (*p* < 0.001). Mg and Sr content was significantly lower in rye-fed groups compared to the other groups (*p* < 0.001, in both cases). Xylanase supplementation had a significant effect on P (*p* < 0.01), Fe (*p* < 0.001), and Cu (*p* < 0.01), only in the rye–corn–wheat group, increasing the content of these minerals in the eggshells. No differences in Na, K, or Zn content in the eggshells were observed between treatments (*p* > 0.05).

The eggshell mechanical properties are presented in Table 4 and Table 5. Generally, no differences between treatments in eggshell mechanical properties were observed, irrespective of the egg plane in which the compression test was applied (*p* > 0.05). The only differences between treatments were observed with regards to the level of egg deformation at which cracking occurred. Supplementation with xylanase to hens fed the wheat–corn diet decreased the cracking deformation level of eggs in the polar plane (*p* < 0.05, Table 4). The opposite effect was observed when eggs were compressed in the equator plane (Table 5), where xylanase supplementation had no effect on deformation rate of eggs from hens fed the corn–wheat diets and it decreased the cracking deformation of eggs from hens fed the rye–corn–wheat diet (*p* < 0.05).

### 3.2. Bone Properties

Tibia morphological properties and densitometric data are presented in Table 6. While tibia weight was not different between treatments (*p* > 0.05), hens fed the rye–corn–wheat diets had longer tibias (*p* < 0.05) with a thicker bone diaphysis, as indicated by significantly increased values for mid-diaphysis cross-sectional area (*p* < 0.001) and cortical index (*p* < 0.001). The changes in bone geometry resulted in significantly decreased values of BMD and BMC (*p* < 0.01 in both cases) of bones from hens fed the rye–corn–wheat diets. There was also a significant effect of enzyme supplementation observed with regards to some bone traits. The tibias of hens fed xylanase supplemented diets were shorter (*p* < 0.05), with a greater cortical index in the bone midshaft area (*p* < 0.01) and significantly increased values of BMD (*p* < 0.01) and BMC (*p* < 0.05), when compared to the tibias of hens from corresponding xylanase-deprived groups. Xylanase supplementation also increased bone ash percentage of the tibias of hens fed the corn–wheat diet (*p* < 0.001).

Table 7 presents results of the analysis of the chemical composition of the tibia mineral phase. Xylanase supplementation, irrespective of the diet, significantly increased the mineral content of several macro- and microelements, including Ca, Mg, Mn and Cr (*p* < 0.001 in all cases). As a result of the increase in Ca content, accompanied by no changes in P content (*p* > 0.05), an increase in Ca/P ratio in xylanase-supplemented groups was observed (*p* < 0.05). Enzyme supplementation also increased bone Cr content in hens fed the corn–wheat diet (*p* < 0.01).

Xylanase addition, irrespective of the diet, had a positive effect on bone strength, as both yield load and ultimate load were increased in the enzyme-supplemented groups (*p* < 0.01 in both cases, Table 8). Inclusion of enzyme in the wheat–corn diet also significantly increased the value of the total work needed to break the tibia from hens fed the corn-wheat diet (*p* < 0.001). There was no effect of treatments on the bone material properties (*p* > 0.05, Table 9).

## 4. Discussion

Our study investigated the effects of inclusion of a modern hybrid rye and xylanase supplementation to wheat–corn based diets, examining in detail the relationship between eggshell and bone mechanical strength and quality indices at the end of the laying cycle. In-depth analysis of eggshell quality and the hens’ bones during this stage of laying, when the hens are especially susceptible to osteoporotic bone fractures, shows how the overall bone calcium reserves were maintained during the whole egg laying cycle in the different dietary treatment groups.

The results obtained, showed that neither the dietary inclusion of 25% hybrid rye nor the xylanase supplementation for 25 weeks had any effects on egg weight or geometry at the end of the experimental period. Lázaro et al. [32] previously showed that when hens fed cereal-based diets (wheat or rye) are supplemented with a xylanase-containing enzyme complex, egg weight was not different compared to those not receiving the enzyme complex. Eggshell quality traits were, however, affected by the dietary treatments in the present study. While eggshell percentage decreased in rye-fed groups, the values were still within normal ranges with regards to quality standards for the eggshells of commercial layers at this age [33]. Similarly, xylanase addition had no effect on egg weight or geometry, which is consistent with previous work in which xylanase supplementation to wheat–corn based diets did not affect general egg parameters (weight and shape index) [34]. However, xylanase positively influenced eggshell thickness, but only in eggs from hens fed the rye–wheat–corn diet, while the effect on eggshell density was only observed in eggs from layers fed the wheat–corn diet. These results are generally in agreement with previous studies, such as that by Mikulski et al. [35] which also showed that when diets containing hybrid rye (up to 20%) are supplemented with NSP-degrading enzymes, no significant effect on eggshell thickness is observed. Our results agree with other previous studies that have shown no effect of xylanase on eggshell weight or thickness in eggs from hens fed a wheat-based diet alone [36,37] or diets with 10% rye inclusion [38].

The observed changes in eggshell density following xylanase supplementation in the current study probably result, to some extent, from changes in the mineral composition of the eggshell. Hens fed the rye-containing diet responded better to the xylanase supplementation, with increased eggshell mineralization, not only with regards to Ca content, but also that of P, Fe and Cu, which are important for eggshell formation and integrity [39]. P is required for eggshell formation as it is involved in the termination of calcification [40]. Cu is one of the microelements that participates in the formation of eggshells as an enabler of carbonic anhydrase, the action of which is crucial for the proper deposition and orientation of calcine crystals, which could affect eggshell porosity and mechanical strength; it also catalyzes the formation of the eggshell membrane structure through lysyl oxidase [41,42]. Fe, the eggshell content of which was increased in the rye-fed group after xylanase supplementation, mainly affects the eggshell color by regulating the transport of protoporphyrin IX [43]. The eggshell content of Fe can change with changes in the dietary concentration of Fe [44]. The decreased Mg and Sr content in the eggshell of eggs from hens fed the rye diets, is probably a result of the different content of these minerals in rye and wheat grains. Due to high chemical similarity of Sr to Ca, Sr easily substitutes Ca in mineralized structures of bone and eggshell [45], while the presence of Mg in the eggshell results from its role in the formation rate of calcite crystals [40,46]. However, as the actual content of these minerals in grains was not analyzed, this topic requires further investigation.

For old rye varieties, the increased viscosity of the chyme, promoted by the NSPs, leads to decreased digestion of minerals [16,47]. Lei et al. [48] suggested that the effectiveness of xylanase in improving eggshell quality may be primarily associated with improvements in the solubility and absorption of minerals, especially Ca, but also other microelements. In this context, the increase in eggshell mineralization observed in eggs from hens fed the diet containing the modern hybrid rye variety following xylanase supplantation, seems to be very promising. Therefore, the changes in eggshell mineral composition in laying hens fed hybrid rye-containing diets, in response to xylanase, is clearly a topic which requires further investigation and a detailed analysis evaluating the intestinal absorption rates of various minerals may be merited.

To the best of our knowledge, there are no studies on the mechanical properties of the eggshell of eggs from hens fed rye-based diets. Therefore, to thoroughly investigate the effect of dietary inclusion of modern hybrid rye on the eggshell’s strength, a compression test along two egg axes was performed, since eggshell mechanical and structural properties, as well as its susceptibility to cracks and damage, is different for eggs loaded along the X axis (between the poles of the eggs) than for eggs loaded along the Z axis (in the equatorial plane) [25,30]. Yet, no effect of rye inclusion was observed, irrespective of the egg plane in which the compression test was applied.

The effect of xylanase supplementation was limited to the egg deformation rate only. Previous studies in hens at a similar post-peak laying period (46–62 weeks of age) as those of the present study [11,37,48] fed wheat-based diets, as well as younger hens (29–33 weeks of age) fed wheat–corn diets [36], showed no effects of xylanase on the general strength of the eggshell, despite the fact that it is generally believed that older hens respond to xylanase supplementation to a lesser extent than younger hens, since NSPs are better tolerated by older birds with a mature digestive system [34,38]. Although eggshell mineralization and thickness are the main factors contributing to general eggshell strength, thicker eggshells do not guarantee stiffer or stronger eggs, as eggshell ultrastructure is also closely related to eggshell mechanical properties [49]. Solomon [50] suggested that the organization of the columns of the palisade layer is one of the major determinants of the rigidity of eggshell, as well as its resistance to deformation. Additionally, as mentioned above, egg deformation under a load in different egg planes was the only mechanical parameter that changed following xylanase supplementation in the current study. Therefore, it could be assumed that the observed effect of enzyme supplementation to different diets on egg deformation might have been due to changes in the mammillary layer and eggshell microstructure [51,52]. This should be verified by future studies.

The average hen requires over 2 g of Ca for each eggshell and up to 36% of the required Ca used for eggshell formation originates from the bones. While medullary bone serves as a labile calcium store for laying hens, both medullary and structural bones continue to be resorbed during the laying period, which may lead to osteoporosis and bone fractures in the hens [4,53]. The tibia is considered a model bone for the examination of bone health in all poultry species, including laying hens, despite the fact that the femur is the most labile source of bone calcium from medullary bone [54,55]. Both main dietary factors examined in this study influenced tibia geometry. Hens fed rye-containing diets had longer tibias. However, tibia length has been proposed as an indicator of structural body size in birds, including commercial strains of laying hens, rather than an indicator of bone Ca storage capacity [54,56]. From the point of view of Ca homeostasis, more interesting is the fact that both dietary factors increased the thickness of the bone mid-diaphysis, i.e., cortical index (rye and xylanase) and cross-sectional area (rye), the bone region in which medullary bone is formed. Xylanase supplementation also increased the BMD of the whole tibia, when only the bone mid-diaphysis was considered, as reported previously [18], the positive effect of the enzyme revealed itself only in the rye-fed group, which was attributed to the increased mineralization of the medullary bone.

Both DXA measurements and bone ash percentage can be used as indicators of bone mineralization. However, Baird et al. [57] found that DXA underestimated BMC of excised tibias from 58-wk-old hens compared to bone ash. Therefore, bearing in mind the fact that the DXA method estimates the bone mineral content based on 2D scans of the bone, the results of bone ash quantification seem to be a more reliable indicator of bone mineralization. In the current study, we found that xylanase supplementation increased the bone ash percentage of the whole tibia in hens fed the wheat–corn diet, which indicates an improvement in overall bone mineralization. These changes in overall bone mineralization could be related both to an increase in medullary bone formation, or a decrease in compact bone resorption [4]. Nevertheless, similar results were previously observed in the bone mid-diaphysis region, both for bone ash measurements, as well as FT-IR spectroscopy analysis of the bone mineral phase [18].

Bone development and quality depends on the amount of absorbed nutrients and bone turnover rate. Our study showed that dietary rye inclusion has no effect on the mineral composition of the bone from hens at the end of the laying period, while enzyme supplementation positively influenced not only Ca content, crucial for eggshell formation, but also the content of numerous key microminerals. Bones are the main reservoir of Mg, which is a cofactor for numerous enzymes and in bones it also plays a role in hydroxyapatite crystal formation, preventing osteoporosis [58]. Mn stimulates the synthesis of the bone matrix and improves bone regeneration [59]. This once again confirms the anti-osteoporotic action of xylanase. Enzyme inclusion also increased the Ca:P ratio, but all obtained values were within the normal range of values reported for layers at this age [60]. Similar to the improvements in eggshell mineralization, the increased bone mineral content observed in the current study may be due to improved absorption of minerals from the intestine, as a result of the lowered chyme viscosity after xylanase supplementation [48]. Our results are in partial agreement with Olgun et al. [11] who showed that enzyme inclusion in a wheat–corn diet increased tibial Ca content with no effect on P and Zn. However, in contrast to our study, no differences in bone Mg and Mn content were noted. The observed discrepancies between studies are most probably due to differences in the composition of the diets, the age of the laying hens, and in the concentrations and activity of xylanase [48]. To the best of our knowledge, there are no studies examining bone mineralization in hens fed rye-based diets. A decrease in tibia Ca and P content was observed in broiler chickens fed rye, when compared with chickens fed the corn diet [61], but the results from broilers cannot be directly transferred to laying hens.

The three-point bending test was performed to examine the effect of hybrid rye inclusion and xylanase supplementation in the wheat–corn diet. When both diets were supplemented with enzyme, significant improvements in bone mechanical strength were observed, since both the values of yield load, limiting the range of bone elastic deformation, and ultimate load, representing the bone endurance against breaking, were increased. In a previous study by our lab, involving the histological analysis of the maturation of collagen cross-links, it was concluded that xylanase increased the mineralization of medullary and compact bone in hens fed both wheat–corn and rye–wheat–corn diets and this may indicate increased bone strength in xylanase supplemented groups [18]. This was confirmed by the bone mechanical tests in the current study. However, in contrast to our results, Silversides et al. [62] showed that supplementation of xylanase in a wheat–corn diet did not affect the mechanical strength of the humerus or radius of laying hens at 64 weeks of age.

As for the hybrid rye, it can be concluded that the predisposition of the tibia bone mid-diaphysis to fractures, at the end of the laying period, was not affected by dietary rye inclusion. According to Whitehead and Fleming [63], the similar bone breaking strength observed between dietary treatments may indicate similar mobilization of Ca for eggshell formation from the strength-providing cortical bone, since the medullary bone contributes to bone strength to a much lesser extent [64]. Our findings also support the hypothesis that the geometry of the bone only has a limited influence on raw bone breaking strength in hens [65], as discussed above, there were significant differences in tibia cross-sectional shape in rye-fed groups which did not influence bone breaking strength. Finally, when the results of the mechanical tests were expressed in terms of bone material traits, which characterize mechanical properties at the tissue level, no differences between dietary treatments were noted. The lack of differences in bone material levels is supported by our previous findings, which showed that there was no effect of the examined dietary treatments on bone hydroxyapatite crystal sizes, or basal inorganic components of the bone tissue [18]. In that study, we also concluded, based on the organization of the bone organic phase, that the diets assessed should have no effect on bone stiffness. This was also confirmed by the present study results.

## 5. Conclusions

In conclusion, the current study indicates that modern hybrid rye grain can be introduced, along with wheat, to corn-based diets of laying hens, as an energy source at a level of up to 25%, without compromising eggshell quality at the end of the laying period. Xylanase supplementation to these diets is also recommended as it results in improvements in eggshell quality and bone mineralization, indicating its positive effect in the overall maintenance of bone mineral reserves during the whole egg laying cycle.

## Figures and Tables

**Table 1:** Composition and calculated nutritional value of experimental diets.

Item	Diet	
	Wheat–Corn ^1^	Rye–Wheat–Corn ^2^
Xylanase “on top” *	0 or 200 mg/kg	0 or 200 mg/kg
Ingredients (g/kg)		
Rye ^3^	0.00	250.00
Wheat	382.30	250.80
Corn	290.00	150.00
Soybean meal	200.00	210.00
Rapeseed oil	15.00	27.00
Limestone	91.00	90.00
Monocalcium phosphate	12.00	12.00
Sodium chloride	3.00	3.00
DL-Methionine	1.10	1.10
L-Lysine hydrochloride	0.60	0.10
Vitamin-mineral premix ^4^	5.00	5.00
Metabolizable energy, MJ/kg ^5^	11.55	
Nutrient composition (g/kg DM) ^6^		
Crude protein	170.00	
Lysine	7.20	
Methionine	3.40	
Calcium	36.00	
Available phosphorus	3.75	
Sodium	1.50	
Chlorine	2.19	

* Xylanase (Ronozyme WX; declared activity of 1000 FXU/g) was added to xylanase-supplemented groups “on top” of the feed formulation. ^1^ The content of anti-nutritional factors in the wheat–corn diet: total NSP, 77.8 g/kg DM; insoluble NSP, 67.0 g/kg DM; arabinoxylans, 33.4 g/kg DM [21]; ^2^ The content of anti-nutritional factors in rye–wheat–corn diet: total NSP, 93.4 g/kg DM; insoluble NSP, 75.9 g/kg DM; arabinoxylans, 40.84 g/kg DM [21]; ^3^ The chemical composition of rye grain included: 9.23% of crude protein, 0.81% of crude fat, 1.46% of crude fiber and 1.51.% of crude ash [22]; ^4^ The premix provided per 1 kg of diet: vitamin A, 10,000; vitamin D3, 2000 IU; vitamin E, 20 IU; vitamin K3, 1.5 mg; vitamin B1, 1.0 mg; vitamin B2, 4.0 mg; vitamin B6, 1.5 mg; vitamin B12, 0.01 mg; Ca-pantothenate, 8.7 mg; niacin, 20.0 mg; folic acid, 0.8 mg; choline chloride, 200.0 mg; manganese, 85.0 mg; zinc, 60.0 mg; iron, 45.0 mg; copper, 8.0 mg; iodine, 1.0 mg; selenium, 0.25 mg; ^5^ Calculated according to [23], as the sum of the metabolizable energy content of the components; ^6^ Calculated according to the chemical composition of feed components.

**Table 2:** Effect of dietary 25% rye inclusion and xylanase supplementation (200 mg/kg of feed) on external properties of eggshell at the end of the laying cycle.

Factors ^1^		Egg Weight g	Eggshell Percentage, %	Eggshell Thickness, µm	Volume, cm^3^	Shape Index, %	Eggshell Density, g/cm^3^
Rye ^2^	Enzyme ^3^						
Treatment ^4^							
-	-	61.1	13.6	459 ^b^	574	77.2	2.52 ^b^
	+	61.8	13.7	451 ^b^	580	76.6	2.48 ^a^
+	-	60.9	13.1	436 ^a^	571	76.9	2.49 ^a^
	+	60.7	13.2	455 ^b^	565	77.9	2.51 ^b^
SEM ^5^		0.88	0.18	7.4	8.0	0.01	0.007
Main factors							
-		61.5	13.7	455	577	76.9	2.50
+		60.8	13.2	446	568	77.4	2.50
	-	61.0	13.4	447	572	77.1	2.51
	+	61.2	13.4	453	573	77.2	2.49
*p*-value							
Rye		0.439	0.008	0.193	0.265	0.337	0.737
Enzyme		0.753	0.778	0.416	0.938	0.729	0.073
Rye × enzyme		0.612	0.918	0.042	0.448	0.130	<0.001

^1^ Number of observations for calculation of treatment effects *n* = 12, for main factors *n* = 24. ^2^ Groups fed wheat–corn diets with (25%) or without (0%) rye inclusion. ^3^ Groups supplemented with or without xylanase enzyme (200 mg/kg of feed). ^4^ Columns with different superscripts (^a,b^) are significantly different (*p* < 0.05). ^5^ SEM = standard error of the means.

**Table 3:** Effect of dietary 25% rye inclusion and xylanase supplementation (200 mg/kg of feed) on eggshell mineralization at the end of the laying cycle.

Factors ^1^		Ca, mg/g	Mg, mg/g	P, mg/g	K, µg/g	Na, µg/g	Sr, µg/g	Fe, µg/g	Zn, µg/g	Cu, µg/g
Rye ^2^	Enzyme ^3^									
Treatment ^4^										
-	-	262 ^d^	3.13	1.049 ^ab^	640	668	165	5.39 ^b^	5.25	0.838 ^ab^
	+	244 ^b^	3.17	0.956 ^a^	700	608	157	5.13 ^ab^	3.61	0.760 ^a^
+	-	239 ^a^	2.67	0.965 ^a^	651	643	145	5.04 ^a^	3.69	0.727 ^a^
	+	250 ^c^	2.73	1.070 ^b^	621	668	146	5.35 ^b^	3.71	0.961 ^b^
SEM ^5^		1.3	0.068	0.0314	26.0	21.6	2.7	0.070	0.486	0.0446
Main factors										
-		253	3.15	1.002	670	638	161	5.26	4.43	0.790
+		244	2.70	1.017	636	656	145	5.20	3.70	0.844
	-	250	2.90	1.007	645	656	155	5.21	4.48	0.783
	+	247	2.95	1.013	661	638	151	5.24	3.35	0.860
*p*-value										
Rye		<0.001	<0.001	0.627	0.196	0.415	<0.001	0.346	0.138	0.317
Enzyme		0.009	0.457	0.865	0.552	0.422	0.103	0.694	0.104	0.089
Rye × enzyme		<0.001	0.993	0.003	0.091	0.053	0.133	<0.001	0.095	0.001

Ca–calcium, Mg–magnesium, P–phosphorus, K–potassium, Na–sodium, Sr–strontium, Fe–iron, Zn–zinc, Cu–copper. ^1^ Number of observations for calculation for treatment effects *n* = 12, for main factors *n* = 24. ^2^ Groups fed wheat–corn diets with (25%) or without (0%) rye inclusion. ^3^ Groups supplemented with or without xylanase enzyme (200 mg/kg of feed). ^4^ Columns with different superscripts (^a,b^) are significantly different (*p* < 0.05). ^5^ SEM = standard error of the means.

**Table 4:** Effect of 25% dietary rye inclusion and xylanase supplementation (200 mg/kg of feed) on eggshell mechanical properties at the end of the laying cycle–mechanical testing in the polar plane.

Factors ^1^		Cracking Force, N	Deformation, %	Work to Cracking, N·mm	Stiffness, N/mm	Firmness, N/mm	Young’s Modulus, GPa	Toughness, N/mm^3/2^
Rye ^2^	Enzyme ^3^							
Treatment ^4^								
-	-	48.9	1.55 ^b^	11.4	166	58.2	15.9	309
	+	49.4	1.39 ^a^	10.8	169	61.9	18.4	342
+	-	47.7	1.39 ^a^	10.3	162	60.7	17.3	328
	+	45.0	1.46 ^ab^	10.1	155	55.0	17.4	328
SEM ^5^		2.36	0.045	0.56	6.8	3.10	0.80	16.1
Main factors								
-		49.1	1.43	11.1	167	60.1	17.2	325
+		46.4	1.47	10.3	156	57.8	17.4	328
	-	48.3	1.47	10.8	164	59.4	16.6	318
	+	47.2	1.42	10.5	159	58.5	17.9	335
*p*-value								
Rye		0.245	0.353	0.133	0.186	0.480	0.794	0.880
Enzyme		0.634	0.304	0.489	0.765	0.751	0.102	0.315
Rye × enzyme		0.495	0.016	0.748	0.462	0.131	0.133	0.318

^1^ Number of observations for calculation of treatment effects was *n* = 12, for main factors *n* = 24. ^2^ Groups fed wheat–corn diets with (25%) or without (0%) rye inclusion. ^3^ Groups supplemented with or without xylanase enzyme (200 mg/kg of feed). ^4^ Columns with different superscripts (^a,b^) are significantly different (*p* < 0.05). ^5^ SEM = standard error of the means.

**Table 5:** Effect of 25% dietary rye inclusion and xylanase supplementation (200 mg/kg of feed) on eggshell mechanical properties at the end of the laying cycle–mechanical testing in the equatorial plane.

Factors ^1^		Cracking Force, N	Deformation, %	Work to Cracking, N·mm	Stiffness, N/mm	Firmness, N/mm	Young’s Modulus, GPa
Rye ^2^	Enzyme ^3^						
Treatment ^4^							
-	-	44.1	2.22 ^a^	13.0	105	44.8	11.3
	+	44.8	2.28 ^ab^	12.8	105	44.5	10.6
+	-	45.7	2.32 ^b^	13.3	103	45.3	10.7
	+	44.3	2.25 ^a^	12.5	105	45.3	11.0
SEM ^5^		1.74	0.030	0.43	6.1	1.75	0.26
Main factors							
-		44.4	2.25	12.9	105	44.6	11.0
+		45.0	2.28	12.9	104	45.3	10.8
	-	44.9	2.28	13.1	104	45.0	11.0
	+	44.5	2.27	12.6	105	44.9	10.8
*p*-value							
Rye		0.757	0.275	0.984	0.902	0.701	0.655
Enzyme		0.830	0.837	0.249	0.903	0.956	0.384
Rye × enzyme		0.561	0.028	0.439	0.840	0.921	0.071

^1^ Number of observations for calculation of treatment effects *n* = 12, for main factors *n* = 24. ^2^ Groups fed wheat–corn diets with (25%) or without (0%) rye inclusion. ^3^ Groups supplemented with or without xylanase enzyme (200 mg/kg of feed). ^4^ Columns with different superscripts (^a,b^) are significantly different (*p* < 0.05). ^5^ SEM = standard error of the means.

**Table 6:** The effect of dietary 25% rye inclusion and xylanase supplementation (200 mg/kg of feed) on hens’ tibial geometric properties at the end of the laying cycle.

Factors ^1^		Weight, g	Length, mm	CI, %	AREA, mm^2^	CSMI, mm^4^	BMD, g/cm^2^	BMC, g	Ash, %
Rye ^2^	Enzyme ^3^								
Treatment ^4^									
-	-	11.2	119	24.5	19.3	137	0.223	2.44	31.7 ^a^
	+	11.2	118	28.9	21.0	127	0.290	3.06	34.7 ^b^
+	-	11.3	122	29.9	21.6	127	0.196	2.13	32.3 ^ab^
	+	11.4	120	34.5	23.9	144	0.221	2.41	29.7 ^a^
SEM ^5^		0.17	0.7	1.53	1.24	11.7	0.0160	0.173	0.71
Main factors									
-		11.2	119	26.7	20.1	132	0.256	2.75	32.2
+		11.3	121	32.2	22.8	136	0.208	2.27	30.0
	-	11.2	121	27.2	20.4	132	0.209	2.28	32.0
	+	11.3	119	31.7	22.4	135	0.255	2.73	32.2
*p*-value									
Rye		0.411	<0.001	<0.001	0.039	0.098	0.005	0.007	0.003
Enzyme		0.591	0.048	0.005	0.111	0.799	0.006	0.013	0.764
Rye × enzyme		0.740	0.705	0.912	0.799	0.255	0.198	0.329	<0.001

^1^ Number of observations for calculation for treatment effects *n* = 12, for main factors *n* = 24. ^2^ Groups fed wheat–corn diets with (25%) or without (0%) rye inclusion. ^3^ Groups supplemented with or without xylanase enzyme (200 mg/kg of feed). ^4^ Columns with different superscripts (^a,b^) are significantly different (*p* < 0.05). ^5^ SEM = standard error of the means. CI = Cortical index, AREA = mid-diaphysis cross-sectional area, CSMI = cross-sectional moment of inertia, BMD = bone mineral density, BMC = bone mineral content.

**Table 7:** Effect of dietary 25% rye inclusion and xylanase supplementation (200 mg/kg of feed) on hens’ tibial mineralization at the end of the laying cycle.

Factors ^1^		Ca, mg/g	P, mg/g	Mg, mg/g	Zn, µg/g	Fe, µg/g	Mn, µg/g	Cu, µg/g	Cr, µg/g	Ca/P
Rye ^2^	Enzyme ^3^									
Treatment ^4^										
-	-	311	139	4.42	252	57.6	7.74	5.78	5.51 ^a^	2.24
	+	320	140	4.81	257	63.8	8.78	5.57	5.79 ^b^	2.28
+	-	314	139	4.44	231	60.4	7.29	6.64	5.51 ^a^	2.26
	+	319	139	4.79	260	62.4	8.64	5.79	5.54 ^a^	2.29
SEM ^5^		1.2	0.6	0.051	8.9	3.84	0.365	0.497	0.038	0.014
Main factors										
-		315	140	4.62	254	60.7	8.16	5.67	5.65	2.26
+		317	139	4.61	245	61.4	7.97	6.21	5.52	2.28
	-	312	139	4.43	242	59.1	7.41	6.21	5.51	2.25
	+	319	140	4.80	258	63.1	8.71	5.68	5.67	2.29
*p*-value										
Rye		0.204	0.384	0.947	0.319	0.848	0.601	0.283	0.002	0.238
Enzyme		<0.001	0.390	<0.001	0.072	0.290	<0.001	0.293	<0.001	0.010
Rye × enzyme		0.106	0.330	0.753	0.186	0.588	0.878	0.523	0.002	0.644

Ca–calcium, P–phosphorus, Mg–magnesium, Zn–zinc, Fe–iron, Mn–manganese, Cu–copper, Cr–chromium. ^1^ Number of observations for calculation for treatment effects *n* = 12, for main factors *n* = 24. ^2^ Groups fed wheat–corn diets with (25%) or without (0%) rye inclusion. ^3^ Groups supplemented with or without xylanase enzyme (200 mg/kg of feed). ^4^ Columns with different superscripts (^a,b^) are significantly different (*p* < 0.05). ^5^ SEM = standard error of the means.

**Table 8:** Effect of 25% dietary rye inclusion and xylanase supplementation (200 mg/kg of feed) on hens’ tibial mechanical properties and geometrical properties at the end of the laying cycle–bone structural properties.

Factors ^1^		Yield Load, N	Ultimate Load, N	Elastic Energy, mJ	Work to Fracture, mJ	Stiffness, N/mm
Rye ^2^	Enzyme ^3^					
Treatment ^4^						
-	-	104	149	21.0	98 ^a^	246
	+	119	182	24.0	259 ^b^	260
+	-	97	156	21.4	106 ^a^	235
	+	110	174	22.8	115 ^a^	274
SEM ^5^		4.8	9.5	1.52	12.8	16.6
Main factors						
-		120	166	22.5	179	253
+		122	165	22.1	111	254
	-	100	152	21.2	102	241
	+	114	178	23.4	187	267
*p*-value						
Rye		0.096	0.992	0.775	<0.001	0.943
Enzyme		0.005	0.009	0.157	<0.001	0.118
Rye × enzyme		0.795	0.455	0.595	<0.001	0.468

^1^ Number of observations for calculation of treatment effects *n* = 12, for main factors *n* = 24. ^2^ Groups fed wheat–corn diets with (25%) or without (0%) rye inclusion. ^3^ Groups supplemented with or without xylanase enzyme (200 mg/kg of feed). ^4^ Columns with different superscripts (^a,b^) are significantly different (*p* < 0.05). ^5^ SEM = standard error of the means.

**Table 9:** Effect of dietary 25% rye inclusion and xylanase supplementation (200 mg/kg of feed) on hens’ tibial mechanical properties and geometrical properties at the end of the laying cycle–bone material properties.

Factors ^1^		Young’s Modulus, MPa	Yield Strain, %	Ultimate Strain, %	Yield Stress, MPA	Ultimate Stress, MPa
Rye ^2^	Enzyme ^3^					
Treatment						
-	-	105	9.82	25.4	11.2	17.3
	+	124	9.49	32.2	13.7	20.8
+	-	93	9.51	24.8	11.1	17.5
	+	98	9.32	24.8	11.2	17.7
SEM ^4^		11.7	0.335	2.57	0.68	1.40
Main factors						
-		138	9.65	28.8	12.4	19.1
+		120	9.41	24.8	11.1	17.6
	-	122	9.67	25.1	11.1	17.4
	+	135	9.40	28.5	12.4	19.3
*p*-value						
Rye		0.121	0.478	0.130	0.060	0.311
Enzyme		0.310	0.431	0.199	0.057	0.185
Rye × enzyme		0.516	0.838	0.194	0.084	0.238

^1^ Number of observations for calculation for treatment effects *n* = 12, for main factors *n* = 24. ^2^ Groups fed wheat–corn diets with (25%) or without (0%) rye inclusion. ^3^ Groups supplemented with or without xylanase enzyme (200 mg/kg of feed). ^4^ SEM = standard error of the means.

## Data Availability

Data are available from the corresponding author upon reasonable request.
